# Is it Time to Consider the “Burnout Syndrome” A Distinct Illness?

**DOI:** 10.3389/fpubh.2015.00158

**Published:** 2015-06-08

**Authors:** Renzo Bianchi, Irvin Sam Schonfeld, Eric Laurent

**Affiliations:** ^1^Institute of Work and Organizational Psychology, University of Neuchâtel, Neuchâtel, Switzerland; ^2^Department of Psychology, The City College of the City University of New York, New York, NY, USA; ^3^Department of Psychology, University of Franche-Comté, Besançon, France

**Keywords:** burnout syndrome, conceptual overlap, depression, depressive disorders, differential diagnosis, mood disorders, nosology, stress

## Abstract

The “burnout syndrome” has been defined as a combination of emotional exhaustion, depersonalization, and reduced personal accomplishment caused by chronic occupational stress. Although there has been increasing medical interest in burnout over the last decades, it is argued in this paper that the syndrome cannot be elevated to the status of diagnostic category, based on (1) an analysis of the genesis of the burnout construct, (2) a review of the latest literature on burnout-depression overlap, (3) a questioning of the three-dimensional structure of the burnout syndrome, and (4) a critical examination of the notion that burnout is singularized by its *job-related* character. It turns out that the burnout construct is built on a fragile foundation, both from a clinical and a theoretical standpoint. The current state of science suggests that burnout is a form of depression rather than a differentiated type of pathology. The inclusion of burnout in future disorder classifications is therefore unwarranted. The focus of public health policies dedicated to the management of “burnout” should not be narrowed to the three definitional components of the syndrome but consider its depressive core.

Burnout has been defined as a combination of emotional exhaustion, depersonalization, and reduced personal accomplishment caused by chronic work stress (1). This constellation of symptoms involves overwhelming fatigue and loss of motivation, a cynical view of one’s job, and a sense of ineffectiveness and failure. Burnout has been presented as an increasingly prevalent phenomenon in modern societies and has received growing attention from both researchers and practitioners, since it was initially described in the 1970s (2–4). Whether burnout should be considered an illness in its own right, however, remains a highly debated issue in the scientific and medical spheres.

While burnout does not appear in the 5th edition of the *Diagnostic and Statistical Manual of Mental Disorders* [(*DSM-5*); (5)], it has been established as a legitimate justification for sick leave in several countries, for instance Sweden (6). Another illustration of the increasing recognition given to the syndrome is burnout’s having been identified as a factor influencing health status and contact with health services in the 10th edition of the *International Classification of Diseases* (*ICD-10*) – burnout is coded Z73.0 and defined as a *state of vital exhaustion* (7). In the present paper, we suggest that, despite the current trend toward using the burnout label as a medical diagnosis (4, 6), the burnout phenomenon is unlikely to represent a differentiated pathological entity. As a corollary, we argue against the elevation of burnout to the status of nosological category in classification systems under preparation.

We enumerate four reasons why burnout should not become a nosological category. First, the foundation on which the burnout construct sits is tenuous. Second, burnout substantially overlaps with depression. Third, the three-dimensional structure of the burnout syndrome is unrealistic. Fourth, the mere fact of defining burnout as job-related is not nosologically discriminant.

## The Burnout Construct is a Fragile Construction

A number of methodological flaws mark the elaboration of the burnout construct and weaken its validity (8). In the original interviews and observations from which the construct of burnout emerged, the presence of already-described stress-related conditions (e.g., depressive syndromes) was not investigated in a systematic manner, suggesting that the conceptualization of burnout symptoms as constituents of a separate entity (1) may be an artifact of a poorly controlled approach to illness characterization (9, 10). In addition, it is noteworthy that the instrument of reference for the assessment of burnout, the Maslach Burnout Inventory [MBI; (1)], is “neither grounded in firm clinical observation nor based on sound theorizing” [(11), p. 3; see also Ref. (8, 12)]. Instead, “it has been developed inductively by factor-analyzing a rather arbitrary set of items” [(11), p. 3; see also Ref. (12), p. 188). The arbitrariness surrounding the elaboration of the MBI constitutes a fundamental problem, especially given the central role of the instrument in the definition of the burnout phenomenon – “burnout is what the MBI measures” [(12), p. 188] – and the growth of burnout research as a whole (8, 11). As noted by some leading investigators in the field of burnout research (12), if other items had been submitted to that original factor analysis, most probably, other dimensions would have emerged, and burnout would have been defined differently. This state of affairs undermines the burnout construct at its foundation.

## Burnout Overlaps with Depression

Depression is primarily defined by anhedonia and dysphoric mood (5). Chronic, unresolvable stress and the impossibility of effective/gratifying action have long been regarded as key depressogenic factors (13–17). Substantiating the aforementioned concerns related to the genesis of the burnout construct, a growing corpus of evidence suggests that burnout problematically overlaps with depression.

Burned out individuals have been found to report as many depressive symptoms as clinically depressed patients, underlining qualitative *and* quantitative overlap of the two entities [see Ref. (8)]. In a 5575-participant study (18), no fewer than 90% of the individuals categorized as burned out met criteria for a provisional diagnosis of depression – as established by the 9-item depression module of Patient Health Questionnaire [PHQ-9; (19)]. A majority of the individuals identified as depressed reported symptoms of *DSM-5* depression *with atypical features* – mood reactivity, significant weight gain or hyperphagia, hypersomnia, leaden paralysis, and interpersonal rejection sensitivity resulting in social or occupational impairment (5).^1^ Interestingly, atypical depression and burnout have both in past research been associated with pervading fatigue, a chronic course, and hypocortisolism (8).

In addition, in a recent eye-tracking study (22), burnout and depression were found to predict similar attentional and behavioral alterations. These alterations consisted of increased focusing on “dysphoric stimuli” and decreased focusing on “positive stimuli.” Burnout and depression were interchangeable in their capacity to predict those alterations. Another recent study has shown that the association between allostatic load – a biological index of the cumulative impact of chronic stress on the organism – and burnout was no longer significant when depression was statistically controlled (23). Finally, in longitudinal studies adopting person-centered approaches (9, 24), burnout and depressive symptoms have been found to be inseparably linked, increasing or declining together over time. Thus, although some researchers have suggested that burnout is irreducible to depression (1, 25), this hypothesis has become less and less plausible as research has advanced. As assumed by Bianchi et al. (9), from a historical standpoint, the burnout construct may reflect a disciplinary divide between (social) psychology and psychiatry, rather than capture a distinct pathological phenomenon.

## The Structure of the Burnout Syndrome is Incoherent

Consistent with the previously addressed points, the basic structure of burnout as a three-dimensional syndrome has been seriously questioned in the last decade (9, 26, 27). In many studies [see Ref. (9)], emotional exhaustion – the core of burnout (1, 12, 28) – has been found to be more strongly associated with depressive symptoms than with the two other definitional dimensions of the syndrome – depersonalization and poor personal accomplishment. Based on such observations, it is unclear why depersonalization and poor personal accomplishment are considered constituents of the burnout syndrome whereas depressive symptoms are not. These findings support the view that the field-dominating definition of burnout is artificial and does not tap a discrete, unified pathological phenomenon.

## Defining Burnout as a Job-Related Syndrome is not Nosologically Discriminant

It has often been claimed that burnout is singularized by its *job-related* character [e.g., Ref. (1)]. However, it should be observed that the attribution of an illness to a specific domain, for instance work, is not nosologically discriminant *per se* (8). A job-related depression (29–31), for example, remains a depression. A new nosological category is not needed on the grounds that domain-specific etiological factors are discernible.

Furthermore, it is worth remembering that the restriction of the definition of burnout to the occupational domain is (1) arbitrary and (2) self-fulfilling when burnout is assessed with the MBI, given that the MBI does not allow for an assessment of emotional exhaustion, depersonalization, and personal accomplishment beyond the job context (32, 33).^2^ In sum, the “job-relatedness” argument does not provide a solid basis for singularizing burnout, notably with respect to depression.

## Conclusion

The conditions under which the burnout construct was elaborated as well as the accumulated evidence on burnout-depression overlap cast doubt on the nosological distinctiveness of burnout. The current state of science suggests that burnout is a form of depression rather than a differentiated type of pathology. Hence, distancing ourselves from the trend toward viewing burnout as a singular illness, we do not recommend the inclusion of burnout as a disorder in upcoming systems of classification.

The focus of public health policies dedicated to the management of “burnout” should not be narrowed to the three definitional components of the syndrome but consider its depressive core. Clinically speaking, treatments for depression offer hope to help individuals identified as “burned out.” Future research should more systematically investigate the environmental contributors to depression in relation to the chronic (work) stress literature in order to propose a more integrative view of this spectrum of disorders and limit the proliferation of redundant or ill-delimited diagnostic categories.

## Conflict of Interest Statement

The authors declare that the research was conducted in the absence of any commercial or financial relationships that could be construed as a potential conflict of interest.

## Footnotes

^1^The term *atypical* “does not connote an uncommon or unusual clinical presentation” [(5); p. 186]. Depression with atypical features is a frequently met form of depression (20, 21).

^2^Remarkably, some minority conceptualizations of burnout do not restrict the syndrome to work (26, 34). However, such conceptualizations have carried little weight in burnout research thus far.
